# Novel Stably Transfected Human Reporter Cell Line AIZ-AR as a Tool for an Assessment of Human Androgen Receptor Transcriptional Activity

**DOI:** 10.1371/journal.pone.0121316

**Published:** 2015-03-26

**Authors:** Iveta Bartonkova, Aneta Novotna, Zdenek Dvorak

**Affiliations:** Department of Cell Biology and Genetics, Faculty of Science, Palacky University, Olomouc, Czech Republic; University of Kentucky College of Medicine, UNITED STATES

## Abstract

Androgen receptor plays multiple physiological and pathological roles in human organism. In the current paper, we describe construction and characterization of a novel stably transfected human reporter cell line AIZ-AR for assessment of transcriptional activity of human androgen receptor. Cell line AIZ-AR is derived from human prostate carcinoma epithelial cell line 22Rv1 that was transfected with reporter plasmid containing 3 copies of androgen response regions (ARRs) followed by a single copy of androgen response element (ARE) from the promoter region of human prostate specific antigen (PSA) gene. AIZ-AR cells remained fully functional for more than 60 days and over 25 passages in the culture and even after cryopreservation. Time-course analyses showed that AIZ-AR cells allow detection of AR ligands as soon as after 8 hours of the treatment. We performed dose-response analyses with 23 steroids in 96-well plate format. We observed activation of AR by androgens, but not by estrogens and mineralocorticoids. Some glucocorticoids and progesterone also induced luciferase, but their potencies were 2-3 orders of magnitude weaker as compared to androgens. Taken together, we have developed a rapid, sensitive, selective, high-throughput and reproducible tool for detection of human AR ligands, with potential use in pharmacological and environmental applications.

## Introduction

Androgen receptor (AR, NR3C4) is a 110-kDa ligand-activated transcriptional factor that belongs to the steroid hormone receptor superfamily. It has broad physiological functions, including developmental and psychological. AR is also involved in several pathological situations, including genesis of prostatic hyperplasia and prostate cancer function or altered pubertal development due to its mutations [1]. In the absence of a ligand, AR primarily resides in the cytoplasm bound to chaperone proteins. Upon activation, AR translocates to the nucleus where it forms AR/AR homodimer, which binds specific DNA sequence known as androgen response element (ARE) and stimulates expression of androgen-responsive genes [2, 3]. Endogenous ligands for AR are testosterone and 5α-dihydrotestosterone (DHT). There is an extensive need for identification of AR ligands, mainly for two reasons. Firstly, AR is a target for several drugs in human pharmacotherapy; therefore, identification and characterization of AR ligands as new lead compounds in drug discovery and development need effective experimental tool. Secondly, various environmental pollutants cause so called endocrine disruption in humans, which occurs often through interactions with steroid receptors signaling, including by AR [4, 5]. Hence, the development of *in vitro* experimental tool for analyses of androgenic and antiandrogenic effects of environmental matrices is of great importance.

Several approaches have been used to assess the effects of foreign compounds and mixtures on transcriptional activity of androgen receptor. In the past, *in vivo* experiments were carried out in rats [6] or transient transfections were performed [4, 7]. Both approaches are costly, time-consuming and they have low capacity for testing (low throughput). Therefore, several stably transfected gene reporter cell lines were introduced to provide reliable and high-throughput method of screening AR transcriptional activity. Térouanne *et al*. (2000) published a construction of prostatic cell line called PALM derived from human prostatic PC-3 cells stably co-transfected with human androgen receptor and reporter plasmid containing firefly luciferase gene under control of androgen-dependent promoter MMTV (mouse mammary tumor virus) [8]. Human breast cancer cell line T47D was used for development of AR-LUX reporter cell line, transfected with reporter plasmid pPBARE2tataluc^+^ containing two copies of the rat probasin androgen-response element. Response of AR-LUX cell line to androgens was maximally five-fold induction to R1881 [9]. MDA-kb2 cell line was constructed by transfection of human breast cancer cell line MDA-MB-453 with reporter gene construct MMTV.luciferase.neo and used for screening of compound for androgenic and glucocorticoid activity [10]. Sonneveld *et al*. (2005) established an androgen receptor gene reporter cell line named AR CALUX derived from human osteosarcoma cell line U2-OS using co-transfection of reporter plasmid 3xHRE-TATA-Luc and expression plasmid pSG5-neo-AR and they tested induction by DHT, maintenance of responsiveness and stability. After that, a panel of structurally and functionally diverse chemicals was tested for androgenic and anti-androgenic effects [11].

In the current paper, we present a novel stably transfected human gene reporter cell line for assessment of AR transcriptional activity. AIZ-AR cell line is derived from human prostate carcinoma epithelial cell line 22Rv1 expressing endogenous AR (no extra AR vector co-transfected) that was transfected with reporter plasmid containing sequence of androgen response element from promoter of human prostate-specific antigen (PSA). AIZ-AR cell line provides a tool for high-throughput (96-well plates) and sensitive identification and characterization of compounds with androgenic and anti-androgenic activity. Cell line AIZ-AR allows detection of androgens as soon as after 8 hours of incubation, and it remains fully functional for more than 28 passages and over 67 days in culture as well as after cryopreservation. Significant advancements and added value for AIZ-AR cell line are demonstrated, as compared to yet developed cell lines, e.g. human AR-LUX [9] and MDA-kb2 lines [10] transfected with reporters containing rodent promoters or human cell lines PALM [8] and AR CALUX [11] over-expressing AR vector.

## Materials and Methods

### Compounds and reagents

DMSO, hygromycin B, testosterone, spironolactone, dexamethasone, beclomethasone, betamethasone, cortisol, corticosterone, aldosterone, prednisolone, methylprednisolone, 17α-progesterone, progesterone, estradiol, diethylstilbestrol and 4-hydroxytamoxifen were purchased from Sigma-Aldrich (Prague, Czech Republic). Danazol, cyproterone acetate, mifepristone, triamcinolone, genistein, raloxifene hydrochloride and tamoxifen citrate salt were from Santa Cruz Biotechnology (Santa Cruz, USA). Fugene HD transfection reagent was purchased from Roche (Basel, Switzerland). Reporter lysis buffer was from Promega (Hercules, CA). All other chemicals were of the highest quality commercially available.

### Cell line

Human prostate carcinoma epithelial cells 22Rv1 (ECACC No. 105092802) were cultured in RPMI-1640 medium supplemented with 10% of fetal bovine serum, 100 U/mL streptomycin, 100 μg/mL penicillin, 4 mM L-glutamine and 1 mM sodium pyruvate. Cells were maintained at 37°C and 5% CO_2_ in a humidified incubator.

### Reporter plasmid

Reporter plasmid p3ARR/ARE-luc2P/minP/hygro was designed as follows: three copies of ARR sequence (CAGGGATCAGGGAGTCTCACA) followed by a single copy of ARE sequence (TGCAGAACAGCAAGTGCTAGC) from promoter region of human prostate-specific antigen (PSA) gene were inserted into pGL4.27 [luc2P/minP/hygro] vector (Cat. No. E8451; Promega, Hercules, CA), using Kpn-1/Xho restriction enzymes.

### Stable transfection of 22Rv1 cells

22Rv1 cells were transfected with reporter plasmid p3ARR/ARE-luc2P/minP/hygro (4 μg) using Fugene HD transfection reagent and seeded at density 8×10^5^ cells in 60 mm culture dishes in 5 mL of the RPMI-1640 medium. Following 48 hours of incubation, the culture medium was replaced by the selection medium supplemented with hygromycin B (0.5 mg/mL). The selection medium was changed every 3–4 days for the period of 3 weeks until a polyclonal population was selected. Subsequently, the cells were transferred to 10 cm culture dishes at the density 300–700 cells per dish and cultured for additional 2 weeks in the selection medium until small colonies were visible. Thereafter, 23 colonies were sub-cloned to 24-well culture plate to obtain monoclonal populations. Clones 8 and 14 were selected for further characterization of resulting transgenic AIZ-AR cells. The use of GMO at Faculty of Science, Palacky University Olomouc was approved by the Ministry of the Environment of the Czech Republic (ref. 91997/ENV/10).

### Cytotoxicity Assay

AIZ-AR cells were seeded in 96-well plates at density 5x10^4^ cells per well. Following 16 h of stabilization, cells were treated with tested compounds and vehicle (DMSO; 0.1% v/v). After 24 h, medium was replaced by charcoal stripped RPMI-1640 medium supplemented with 10% of MTT (10 mg/mL) and incubated for additional 2 hours. Following the incubation, MTT assay was measured spectrophotometrically at 540 nm using Tecan Infinite M2000 plate luminometer.

### Inhibition of firefly luciferase

AIZ-AR cells were treated with 100 nM DHT for 24 hours. Cell lysates containing firefly luciferase, having a catalytic activity corresponding to model ligand-treated cells, were isolated. Tested compounds (10 μM) were added to cell lysates and luciferase activity was measured.

### Gene Reporter Assay

AIZ-AR cells were seeded in 96-well plates at density 5x10^4^ cells per well. Following 16 h of stabilization, cells were treated with tested compounds. After the treatments, cells were lysed and luciferase activity was measured in 96-well plate format using Tecan Infinite M2000 plate luminometer.

### Statistical Analyses

Student’s pair t-test as well as calculations of EC_50_ and IC_50_ values were performed using GraphPad Prism version 6.00 for Windows, GraphPad Software, La Jolla California USA (www.graphpad.com).

## Results

### Generation of stably transfected AIZ-AR clones

**Fig 1:**
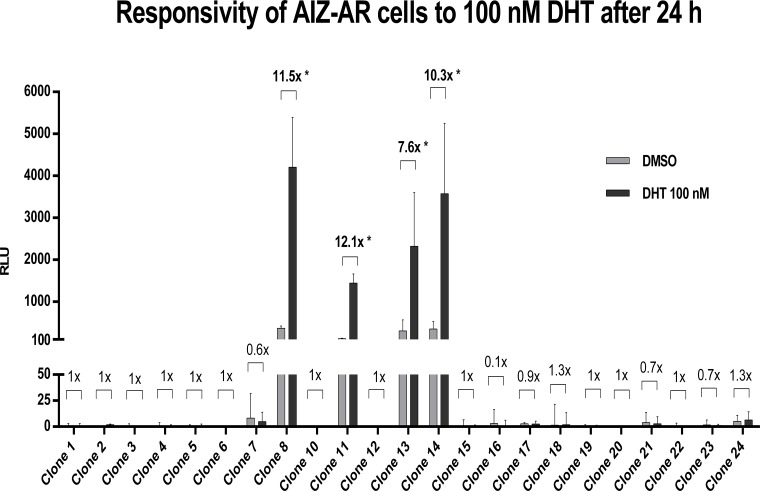
Responsivity of hygromycin-resistant AIZ-AR clones to 100 nM DHT after 24 hours. Cells were treated for 24 h with 5α-dihydrotestosterone (DHT; 100 nM) and vehicle (DMSO; 0.1% v/v). Luciferase activity was measured in cell lysates. Data are mean from triplicate measurements and are expressed as relative luciferase units (RLU). Fold induction over DMSO-treated cells was calculated and it is indicated above each clone in the bar graph. Similar data were obtained from three consecutive passages.

Human prostate carcinoma epithelial cell line 22Rv1 was transfected by lipofection with a reporter plasmid p3ARR/ARE-luc2P/minP/hygro. Stably transfected population of AIZ-AR cells were selected using hygromycin B, as described in Materials and Methods section. We obtained 23 hygromycin B-resistant clones that showed the same morphology as parental 22Rv1 cell line. We tested the responsiveness of clones to 5α-dihydrotestosterone (DHT), a model AR agonist. Cells were treated with DHT (100 nM) and vehicle (DMSO; 0.1% v/v) for 24 hours. Four clones displayed an induction of luciferase activity after the DHT treatment (clones 8, 11, 13 and 14) with fold inductions ranging from 7.6-fold to 12.1-fold (Fig. 1). Clones 8 and 14 were selected for further characterization due to strongest obtained signal, i.e. relative luciferase units (RLU).

### Maintenance of AIZ-AR cells functionality after cryopreservation

**Fig 2:**
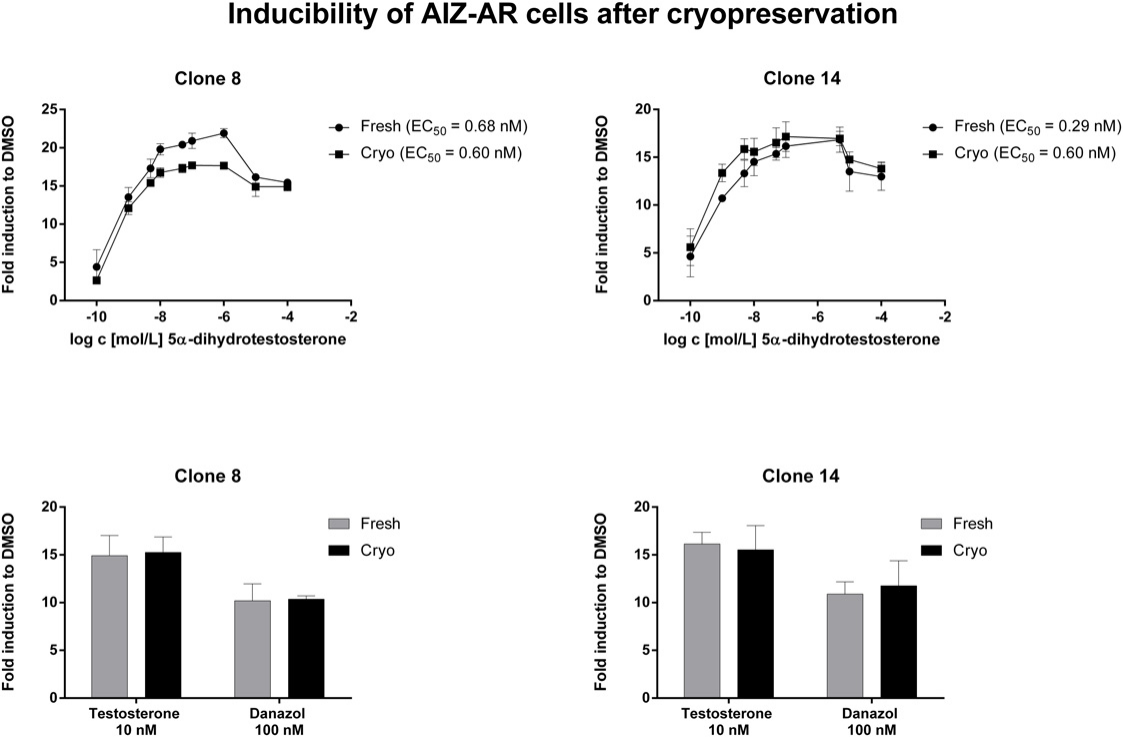
Induction of luciferase activity in AIZ-AR cells after cryopreservation. Cells were treated for 24 h with 5α-dihydrotestosterone (DHT; 0.1 nM—10 μM), testosterone (10 nM) and danazol (100 nM). Cells were lysed and luciferase activity was measured. Data are mean from triplicate measurements and are expressed as fold induction over DMSO-treated cells. Similar data were obtained from three independent experiments (three passages).

We tested the maintenance of luciferase activity induction in AIZ-AR cells after freeze-thaw cycle—cryopreservation. For this purpose, cells were frozen in fetal bovine serum and DMSO as cryo-protectant in ratio 9:1 and stored in -80°C for 1 week. After thawing, both fresh and cryopreserved cells were seeded in 96-well plates at density 5x10^4^ cells per well. Following 16 h of stabilization, cells were treated for 24 h with AR agonists including DHT (0.1 nM-100 μM), testosterone (10 nM) and danazol (100 nM). No significant difference was observed between cryopreserved and fresh cells in terms of fold inductions and EC_50_ values (Fig. 2). Therefore, AIZ-AR cell line can be considered to remain fully functional after cryopreservation.

### Long-term maintenance of luciferase inducibility in AIZ-AR cells

We tested the ability of both clones of AIZ-AR cells to respond to DHT in long-term period. We checked response of cells to DHT (100 nM; 24 h) after each passage of the cells. The induction of luciferase activity by DHT was stable for more than 2 months of AIZ-AR cells in culture, which corresponds to 27–28 passages. Even though there was some variability between passages, there was no systematic decline or decrease in luciferase induction in both absolute RLU values and fold induction magnitude (Table 1).

**Table 1:** Maintenance of responsivness of AIZ-AR cells to 5α-dihydrotestosterone.

Days in culture	Clone 8			Clone 14		
	Passage	RLU	FOLD	Passage	RLU	FOLD
8	4	3368.8	10.4	3	2383.6	10.9
10	5	5039.6	12.4	4	4757.6	10.1
15	7	2489.2	11.2	6	2133.0	11.8
22	9	1759.2	11.1	8	n.d.	n.d.
25	10	2787.5	20.5	9	2297.8	20.5
30	12	2517.2	22.4	11	2185.0	20.7
32	13	3396.4	29.2	12	2127.2	23.2
37	15	3976.5	21.1	14	2121.4	23.9
39	16	4506.3	16.9	15	4517.0	16.8
44	18	4269.3	6.5	17	3470.3	5.7
46	19	4799.0	13.8	18	4023.5	14.8
51	21	3918.0	20.6	20	4011.0	17.3
53	22	6534.5	21.9	21	5746.5	20.3
58	24	5244.3	18.6	23	4787.0	14.4
60	25	2241.8	20.5	24	2044.8	16.6
65	27	3225.8	18.7	26	3499.0	20.3
67	28	4043.5	18.5	27	2485.5	15.5

### Time-course analyses of responsiveness of AIZ-AR cells to DHT treatment

**Fig 3:**
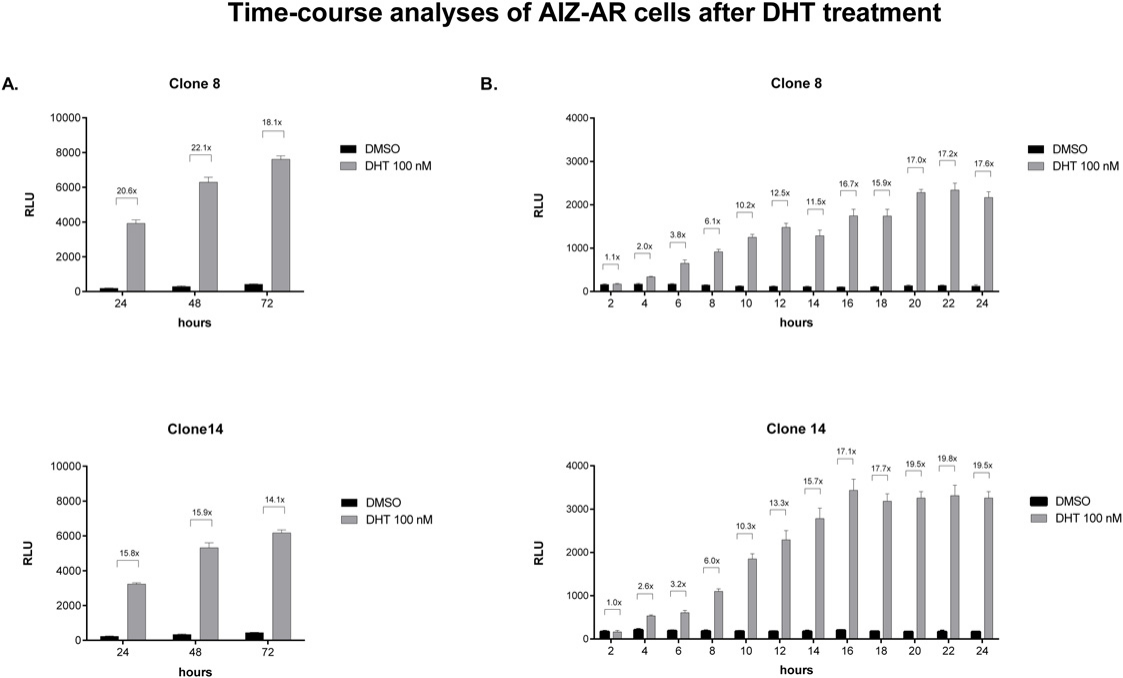
Time-course analyses in AIZ-AR cells after DHT treatment. Cells were treated with 5α-dihydrotestosterone (DHT; 100 nM) and vehicle (DMSO; 0.1% v/v) for time periods from 2 h to 72 h. After the incubations, luciferase activity was measured in cell lysates. Data are mean of triplicate measurements and are expressed as relative luciferase units (RLU). Fold induction over DMSO-treated cells was calculated and it is shown above the bars in the graph. Similar data were obtained from two consecutive passages. Panel A: Long-term time-course analyses; incubation times: 24 h, 48 h and 72 h. Panel B: Short-term time-course analyses; incubation times: 2 h, 4 h, 6 h, 8 h, 10 h, 12 h, 14 h, 16 h, 18 h, 20 h, 22 h and 24 h.

In the first series of experiments, AIZ-AR cells were incubated with DHT (100 nM) for 24 h, 48 h and 72 h. Absolute luciferase activity (RLU) progressively grew with increasing time of incubation and ranged between 4000 and 8000 RLU. However, fold induction over the vehicle-treated cells remained nearly constant, regardless the time of incubation (Fig. 3A). Therefore, incubation of AIZ-AR cells for 24 h is optimal, and prolonged incubation time does not bring additional value. Since evaluation of cytotoxic compounds in cell culture could fail after 24 h of incubation due to the loss of cell viability, we analyzed luciferase induction by DHT in short time periods, in order to find minimal incubation period for reliable identification of AR agonists. For this purpose, we incubated AIZ-AR cells with DHT (100 nM) for 2 h, 4 h, 6 h, 8 h, 10 h, 12 h, 14 h, 16 h, 18 h, 20 h, 22 h and 24 h. We observed time-dependent increase of both RLU and fold induction after the treatment with DHT. Plateau in fold induction was attained approximately after 16 h of incubation. Luciferase activity around 1000 RLU and induction about 6-fold was attained after 8 hours of incubation with DHT, implying the possibility to test cytotoxic compounds in 8 h time period (Fig. 3B).

### Dose-response analyses in AIZ-AR cells treated with a panel of steroids

To assess selectivity of new AIZ-AR cell line towards androgens, we performed dose-response agonist and antagonist analyses using 23 different endogenous and synthetic steroids. Prior to the experiments, we have tested: (i) Cytotoxicity of tested compounds (up to 10 μM) using standard MTT test as described in the methods. Of 23 substances, significant cytotoxicity after 24 h was observed for spironolactone, mifepristone, raloxifene hydrochloride, 4-hydroxytamoxifen and tamoxifen citrate, with IC_50_ values ranging from 1.2 μM to 5.4 μM (Table 2); (ii) Inhibition of firefly luciferase catalytic activity, as described in methods. With exception of genistein, none of tested steroids significantly influenced luciferase activity (data not shown). Overall, effects of tested compound on cell viability and luciferase activity should be taken in account when interpreting the data from gene reporter assays.

**Table 2:** Viability of AIZ-AR cells after treatment with 23 steroid compounds. Data were calculated from triplicate measurements and are expressed as mean ± SD. Analyses were performed in two independent experiments.

Compound	log c/ % of viability					
	-10	-9	-8	-7	-6	-5
5α-dihydrotestosterone	100.0	107.5 ± 4.6	108.7 ± 2.1	114.4 ± 5.7	107.7 ± 2.6	104.1 ± 2.0
Testosterone	100.0	108.6 ± 1.0	108.0 ± 4.8	102.9 ± 5.6	105.4 ± 6.9	105.3 ± 1.0
Danazol	100.0	106.5 ± 1.5	108.1 ± 6.9	109.0 ± 10.6	108.2 ± 11.6	113.6 ± 12.1
Cyproterone acetate	100.0	105.3 ± 1.7	104.7 ± 2.0	105.8 ± 5.7	99.4 ± 7.8	89.5 ± 6.6
Spironolactone	100.0	107.7 ± 1.5	111.8 ± 5.7	112.6 ± 10.0	98.9 ± 5.5	58.9 ± 2.5
Dexamethasone	100.0	99.8 ± 3.9	114.0 ± 2.0	112.7 ± 1.1	111.4 ± 3.4	108.3 ± 2.6
Beclomethasone	100.0	99.9 ± 3.9	106.5 ± 6.1	109.2 ± 5.7	98.4 ± 0.9	92.3 ± 2.1
Betamethasone	100.0	104.9 ± 4.0	110.0 ± 7.3	109.1 ± 7.9	108.0 ± 14.6	101.3 ± 9.9
Cortisol	100.0	109.1 ± 4.1	106.4 ± 9.1	107.3 ± 13.2	107.4 ± 12.3	105.4 ± 10.4
Triamcinolone	100.0	101.0 ± 4.2	100.0 ± 8.2	105.2 ± 11.4	103.2 ± 11.0	101.2 ± 5.4
Prednisolone	100.0	100.7 ± 0.8	103.8 ± 3.7	106.9 ± 5.2	104.2 ± 5.2	101.0 ± 8.3
Methyl-prednisolone	100.0	106.8 ± 3.1	111.4 ± 9.4	104.8 ± 8.9	104.6 ± 14.3	104.9 ± 16.0
Mifepristone	100.0	104.0 ± 2.8	102.2 ± 9.7	103.9 ± 18.2	94.7 ± 9.5	41.7 ± 1.1
Corticosterone	100.0	104.0 ± 0.0	107.3 ± 2.5	110.8 ± 5.7	109.2 ± 6.5	105.2 ± 3.4
Aldosterone	100.0	110.4 ± 1.4	99.4 ± 1.0	99.0 ± 1.4	98.6 ± 0.8	97.0 ± 5.4
17α-progesterone	100.0	98.2 ± 2.6	95.0 ± 4.0	95.0 ± 4.0	92.0 ± 3.7	92.0 ± 3.7
Progesterone	100.0	98.7 ± 7.3	98.7 ± 12.2	99.8 ± 14.1	98.1 ± 13.9	91.0 ± 9.5
17β-estradiole	100.0	106.6 ± 4.3	110.5 ± 3.2	110.2 ± 6.2	110.2 ± 6.2	116.4 ± 16.4
Genistein	100.0	96.5 ± 2.4	96.7 ± 3.3	102.9 ± 7.5	102.9 ± 7.5	119.9 ± 6.4
Diethylstilbestrol	100.0	100.7 ± 5.1	102.1 ± 7.6	101.7 ± 5.1	98.0 ± 4.3	99.9 ± 7.0
4-hydroxytamoxifen	100.0	101.8 ± 1.0	100.0 ± 5.4	103.6 ± 4.4	105.9 ± 3.8	9.2 ± 7.7
Raloxifene hydrochloride	100.0	102.9 ± 8.5	99.8 ± 4.3	105.7 ± 6.8	95.9 ± 10.3	7.1 ± 0.2
Tamoxifen citrate	100.0	102.7 ± 4.3	107.0 ± 4.6	105.5 ± 3.8	88.5 ± 2.4	1.7 ± 0.8

Dose-response analyses assays were performed in two different experimental layouts. In agonist mode, cells were treated with increasing concentrations of tested compounds and EC_50_ (half maximal effective concentration) values were calculated. In antagonist mode, cells were treated with increasing concentrations of tested compounds in combination with model AR agonists DHT (100 nM) or testosterone (10 nM). Where appropriate, IC_50_ (half maximal inhibitory concentration) values were calculated.

**Fig 4:**
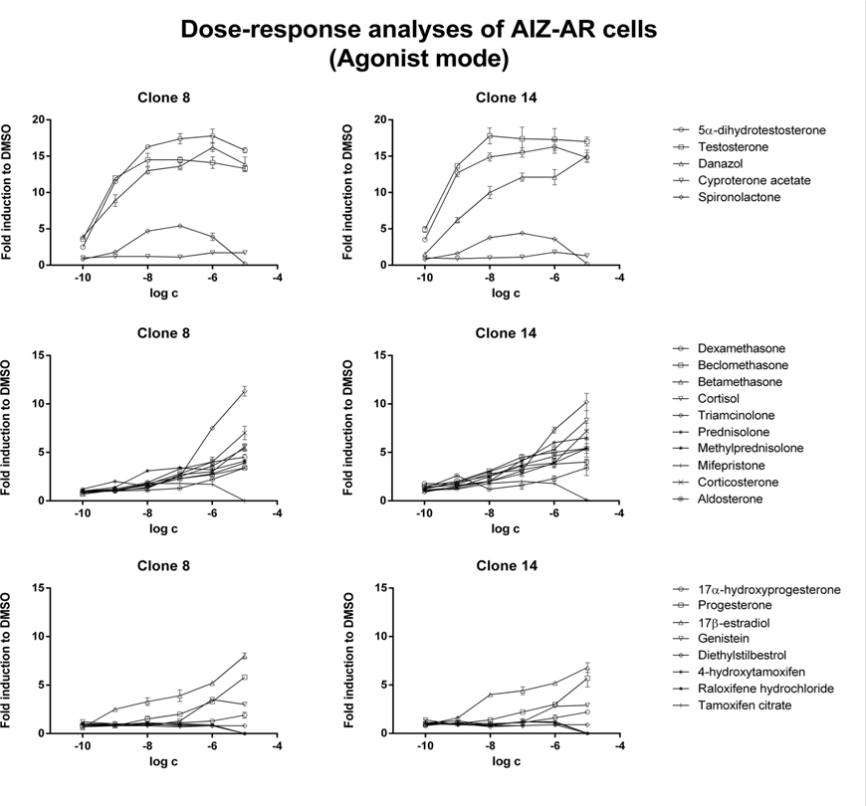
Dose-response analyses in AIZ-AR cells after treatment with steroid compounds—agonist mode. Cells were treated for 24 h with various endogenous and synthetic steroids. Cells were lysed and luciferase activity was measured. Data are mean of triplicate measurements and are expressed as a fold induction over DMSO-treated cells. Similar data were obtained from three consecutive cell passages. Upper plots—androgens, middle plots—corticoids, lower plots—gestagens and estrogens.

In agonist mode, androgens testosterone, DHT and danazol produced typical dose-dependent sigmoid curves. Antiandrogens cyproterone acetate and spironolactone displayed partial agonist and antagonist patterns, respectively (Fig. 4; Table 3). Mineralocorticoid aldosterone and estrogens (with exception of 17β-estradiol) did not significantly increased luciferase activity. Progesterone, 17β-estradiol and some glucocorticoids, in particular triamcinolone, corticosterone and cortisol, also induced luciferase activity, but their potencies were 2–3 orders of magnitude weaker as compared to androgens (Fig. 4; Table 3).

**Table 3:** Characteristics of AIZ-AR cells in comparison with published data. EC_50_—half maximal effective concentration; IC_50_—half maximal inhibitory concentration; n.c.—not calculated; n.d.—not determined.

Compound	Log EC_50_		Log IC_50_ (DHT 100nM)		Log IC_50_ (Testosterone 10 nM)		Literature Log EC_50_	
	Clone 8	Clone 14	Clone 8	Clone 14	Clone 8	Clone 14	Sedlak *et al*.	Wilkinson *et al*.
5α-dihydrotestosterone	-9.16 ± 0.09	-9.19 ± 0.10	-	-	> -5.00	> -5.00	-11.38 ± 0.21	-9.52
Testosterone	-8.92 ± 0.33	-9.10 ± 0.09	> -5.00	> -5.00	-	-	-10.00 ± 0.26	-11.37
Danazol	-8.29 ± 0.34	-8.13 ± 0.12	> -5.00	> -5.00	> -5.00	> -5.00	-9.38 ± 0.17	-9.47
Cyproterone acetate	-6.69 ± 0.17	-6.65 ± 0.51	-6.75 ± 0.05	-6.62 ± 0.02	-7.50 ± 0.04	-7.52 ± 0.06	-8.02 ± 0.19	> -5.00
Spironolactone	-8.80 ± 0.41	-8.36 ± 0.26	-6.37 ± 0.10	-6.17 ± 0.19	-6.72 ± 0.12	-6.65 ± 0.04	-7.52 ± 0.12	> -5.00
Dexamethasone	-7.58 ± 0.31	-7.59 ± 0.27	n.c.	n.c.	n.c.	n.c.	> -5.00	> -5.00
Beclomethasone	-7.59 ± 0.41	-7.70 ± 0.10	n.c.	n.c.	n.c.	n.c.	> -5.00	n.d.
Betamethasone	-6.62 ± 0.04	-7.15 ± 0.26	n.c.	n.c.	n.c.	n.c.	> -5.00	n.d.
Cortisol	-6.35 ± 0.11	-6.49 ± 0.04	n.c.	n.c.	n.c.	n.c.	> -5.00	-8.22
Triamcinolone	-6.37 ± 0.13	-6.56 ± 0.19	n.c.	n.c.	n.c.	n.c.	> -5.00	n.d.
Prednisolone	-6.61 ± 0.09	-7.10 ± 0.38	n.c.	n.c.	n.c.	n.c.	n.d.	n.d.
Methyl-prednisolone	-7.60 ± 0.43	-7.48 ± 0.30	n.c.	n.c.	n.c.	n.c.	n.d.	-8.75
Mifepristone	-5.81 ± 0.05	-5.86 ± 0.06	-6.15 ± 0.10	-6.08 ± 0.03	-6.88 ± 0.20	-6.95 ± 0.21	-7.89 ± 0.13	> -5.00
Corticosterone	-6.22 ± 0.08	-6.25 ± 0.11	n.c.	n.c.	n.c.	n.c.	> -5.00	-8.22
Aldosterone	-6.03 ± 0.04	-6.11 ± 0.02	n.c.	n.c.	n.c.	n.c.	> -5.00	> -5.00
17α-progesterone	-6.45 ± 0.16	-6.29 ± 0.03	> -5.00	> -5.00	> -5.00	> -5.00	> -5.00	n.d.
Progesterone	-6.13 ± 0.08	-6.22 ± 0.12	> -5.00	> -5.00	> -5.00	> -5.00	-7.85 ± 0.11	-7.55
17β-estradiole	-7.19 ± 0.20	-7.54 ± 0.01	> -5.00	> -5.00	> -5.00	> -5.00	-7.30 ± 0.25	> -5.00
Genistein	-6.62 ± 0.42	-6.79 ± 0.23	n.c.	n.c.	n.c.	n.c.	> -5.00	> -5.00
Diethylstilbestrol	> -5.00	> -5.00	> -5.00	> -5.00	> -5.00	> -5.00	> -5.00	> -5.00
4-hydroxytamoxifen	> -5.00	> -5.00	> -5.00	> -5.00	> -5.00	> -5.00	> -5.00	> -5.00
Raloxifene hydrochloride	> -5.00	> -5.00	> -5.00	> -5.00	> -5.00	> -5.00	> -5.00	> -5.00
Tamoxifen citrate	> -5.00	> -5.00	> -5.00	> -5.00	> -5.00	> -5.00	> -5.00	n.d.

Data were calculated from triplicate measuments and are expressed as mean ± SD. Analyses were performed in three independent experiments for each clone.

**Fig 5:**
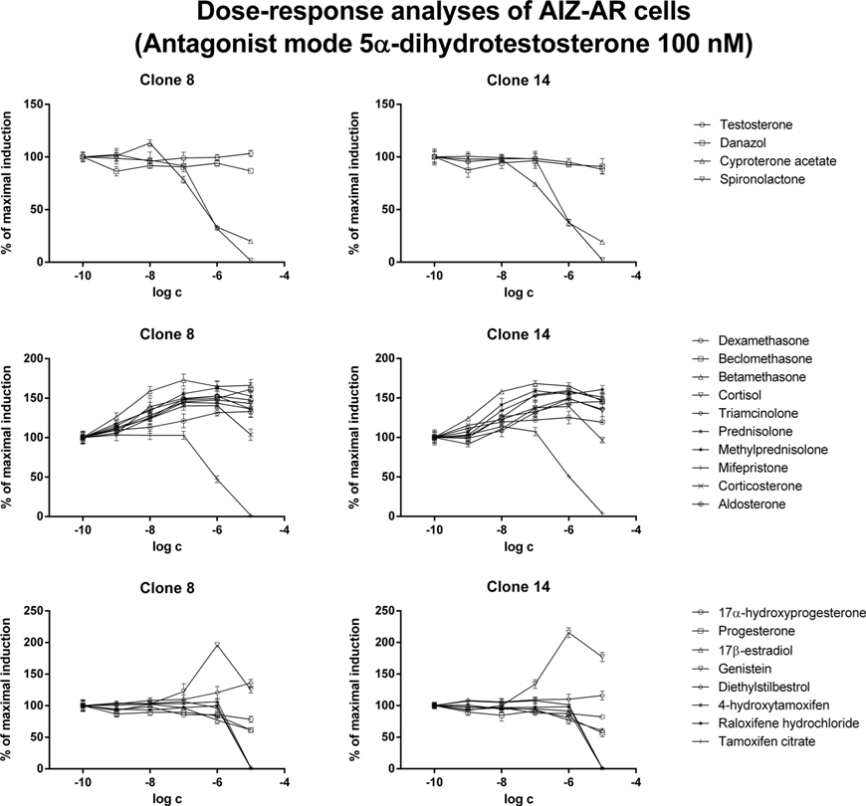
Dose-response analyses in AIZ-AR cells after treatment with steroid compounds—antagonist mode with dihydrotestosterone. Cells were treated for 24 h with various endogenous and synthetic steroids in the presence of 5α-dihydrotestosterone (DHT; 100 nM). Cells were lysed and luciferase activity was measured. Data are mean of triplicate measurements and are expressed as a fold induction over DMSO-treated cells. Similar data were obtained from three consecutive cell passages. Upper plots—androgens, middle plots—corticoids, lower plots—gestagens and estrogens.

**Fig 6:**
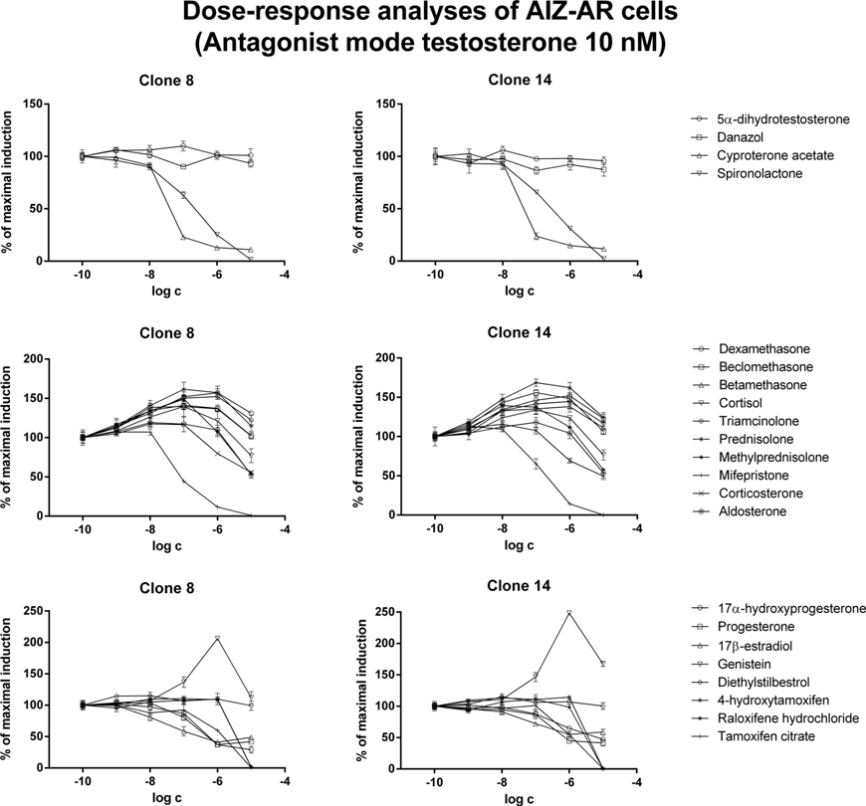
Dose-response analyses in AIZ-AR cells after treatment with steroid compounds—antagonist mode with testosterone. Cells were treated for 24 h with various endogenous and synthetic steroids in the presence of testosterone (10 nM). Cells were lysed and luciferase activity was measured. Data are mean of triplicate measurements and are expressed as a fold induction over DMSO-treated cells. Similar data were obtained from three consecutive cell passages. Upper plots—androgens, middle plots—corticoids, lower plots—gestagens and estrogens.

In antagonist mode, antiandrogens cyproterone acetate and spironolactone dose-dependently inhibited testosterone- and/or DHT-induced luciferase activity. Interestingly, the IC_50_ values for these antiandrogens were approximately 10 times higher in DHT-treated cells as compared to testosterone-treated cells (Fig. 5, Fig. 6; Table 3). Corticoids (except mifepristone, which caused dose-dependent inhibition) yielded an additive effect in concentrations up to 10^-6^ M, resulting in augmentation of luciferase activity in comparison to agonist itself (DHT or testosterone). Interestingly, in concentration of 10^-5^ M, luciferase activity remained augmented when DHT was used as agonist, while it dropped to 60%- 120% of initial agonist-induced activity, when testosterone was used as agonist (Fig. 5, Fig. 6; Table 3).

Inhibitory effects of some estrogens and gestagenes were rather due to their intrinsic cytotoxicity than due to receptor antagonism itself. Interestingly, genistein strongly augmented agonist-induced luciferase activity, with massive drop at 10^-5^ M, which is probably due to the luciferase inhibition. Collectively, AIZ-AR cell line allows effective detection of compounds with androgenic activity. Some cross-reactivity with glucocorticoids occurs in concentrations of 2–3 orders of magnitude higher as compared to androgens. In antagonist experimentation, different profiles depending on the agonist used, possible cytotoxicity and luciferase inhibition must be taken in account when interpreting the data.

## Discussion

In the current paper, we present a novel stably transfected human gene reporter cell line for assessment of human AR transcriptional activity. Human AIZ-AR cell line expresses endogenous functional human AR and it was transfected with reporter plasmid containing sequence of androgen response element from promoter of human prostate-specific antigen (PSA). AIZ-AR cell line provides a tool for high-throughput and sensitive identification and characterization of compounds with androgenic and anti-androgenic activity. Cell line AIZ-AR allows detection of androgens as soon as after 8 hours of incubation, and it remains fully functional for more than 28 passages and over 67 days in culture as well as after freeze/thaw cycle.

Reliable, rapid, sensitive, selective and high throughput tools for assessment of transcriptional activities of nuclear receptors, steroid receptors and xenoreceptors, are needed for various purposes. Drugs targeting androgen receptor are widely used in human pharmacotherapy, e.g. non-steroid antiandrogen flutamide for the treatment of prostate cancer. Therefore, *in vitro* tool for identification and characterization of synthetic androgens and antiandrogens in the process of drug design and development is of value. Since androgen receptor active substances influence hormonal homeostasis, they are referred as to endocrine disruptors. Indeed, there are numerous reports on the use of gene reporter assays in environmental [12], cosmetics [13] or food safety applications [14].

Experimental models differ in their complexity and species-specificity, which has an impact on the reliable and credible transfer of the data to human pharmacology and toxicology. Besides the properties indicated above, the major strengths of AIZ-AR cell line presented here are: (i) AIZ-AR cell line is an exclusively human system; i.e. human maternal cell line, containing endogenous human receptor AR, stably transfected with reporter gene driven by binding sequence from human gene. (ii) AIZ-AR cell line conserves cell signaling stoichiometry; since AIZ-AR cell line contains endogenous human AR, without extra co-transfected AR vector, the stoichiometric ratio between the AR receptor protein and other transcriptional regulators reflects natural situation rather than artificial one with over-expressed AR. The characteristics given above clearly demonstrate significant advancements and added value for AIZ-AR cell line, as compared to yet developed cell lines. Indeed, existing experimental models, such as human AR-LUX [9] and MDA-kb2 lines [10] were transfected with reporters containing rodent promoters but not human ones. In addition cell lines human PALM [8] and AR CALUX [11] are transfected with exogenous AR, therefore, over-expressing AR vector.
